# Diazido­{(*S*)-1-phenyl-*N*,*N*-bis­[(2-pyrid­yl)meth­yl]ethanamine}­copper(II)

**DOI:** 10.1107/S1600536811021234

**Published:** 2011-06-11

**Authors:** Sankara Rao Rowthu, Jong Won Shin, Seung-Hui Kim, Jong Jin Kim, Kil Sik Min

**Affiliations:** a Department of Chemistry, Kyungpook National University, Daegu 702-701, Republic of Korea; b Department of Applied Chemistry, Kyungpook National University, Daegu 702-701, Republic of Korea; c Department of Chemistry Education, Kyungpook National University, Daegu 702-701, Republic of Korea

## Abstract

In the title compound, [Cu(N_3_)_2_(C_20_H_21_N_3_)], the Cu^II^ ion is coordinated by the three N atoms of the (*S*)-1-phenyl-*N*,*N*-bis­[(2-pyrid­yl)meth­yl]ethanamine ligand and two N atoms from two azide anions, resulting in a distorted square-pyramidal environment. A weak inter­molecular C—H⋯N hydrogen-bonding inter­action between one pyridine group of the ligand and an azide N atom of an adjacent complex unit gives a one-dimensional chain structure parallel to the *c* axis.

## Related literature

For the potential applications of chiral complexes in chiral recognition, chiral catalysis and enanti­oselective sorption, see: Lehn (1995 ▶); Seo *et al.* (2000 ▶). Chiral Ni^II^ macrocyclic complexes and two-dimensional chiral open-framework compounds have been described by Han *et al.* (2008 ▶); Ryoo *et al.* (2010 ▶). A homochiral metal–organic framework with a cerium(III) ion has been described by Dang *et al.* (2010 ▶). For the preparation of (*S*)-1-phenyl-*N*,*N*-[bis­(2-pyrid­yl)meth­yl]ethanamine, see: Lucas *et al.* (2009 ▶).
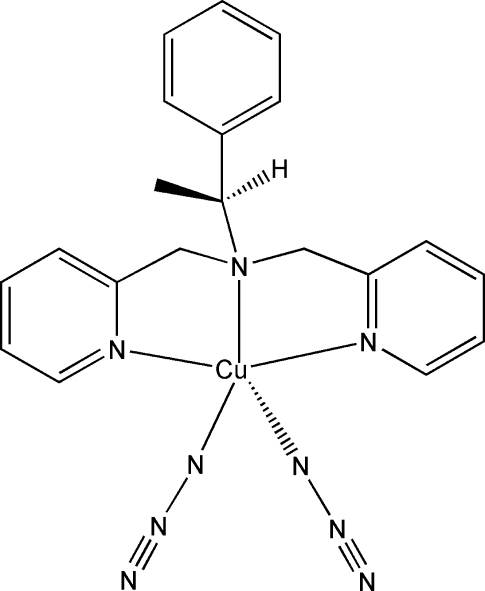


## Experimental

### 

#### Crystal data

[Cu(N_3_)_2_(C_20_H_21_N_3_)]
*M* *_r_* = 451.00
Monoclinic, 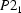

*a* = 6.9972 (12) Å
*b* = 14.506 (3) Å
*c* = 10.2828 (17) Å
β = 98.413 (4)°
*V* = 1032.5 (3) Å^3^
*Z* = 2
Mo *K*α radiation
μ = 1.09 mm^−1^
*T* = 296 K
0.23 × 0.19 × 0.04 mm

#### Data collection

Siemens SMART CCD diffractometer
Absorption correction: multi-scan (*SADABS*; Sheldrick, 1996 ▶) *T*_min_ = 0.749, *T*_max_ = 0.958
7801 measured reflections
4630 independent reflections
2863 reflections with *I* > 2σ(*I*)
*R*_int_ = 0.049

#### Refinement

*R*[*F* ^2^ > 2σ(*F* ^2^)] = 0.058
*wR*(*F* ^2^) = 0.115
*S* = 1.09
4630 reflections
272 parameters
1 restraint
H-atom parameters constrained
Δρ_max_ = 0.74 e Å^−3^
Δρ_min_ = −0.90 e Å^−3^
Absolute structure: Flack (1983 ▶), 1941 Friedel pairs
Flack parameter: 0.02 (3)

### 

Data collection: *SMART* (Siemens, 1996 ▶); cell refinement: *SAINT* (Siemens, 1996 ▶); data reduction: *SHELXTL* (Sheldrick, 2008 ▶); program(s) used to solve structure: *SHELXS97* (Sheldrick, 2008 ▶); program(s) used to refine structure: *SHELXL97* (Sheldrick, 2008 ▶); molecular graphics: *ORTEP-3 for Windows* (Farrugia, 1997 ▶); software used to prepare material for publication: *SHELXL97*.

## Supplementary Material

Crystal structure: contains datablock(s) global, I. DOI: 10.1107/S1600536811021234/pk2326sup1.cif

Structure factors: contains datablock(s) I. DOI: 10.1107/S1600536811021234/pk2326Isup2.hkl

Additional supplementary materials:  crystallographic information; 3D view; checkCIF report

## Figures and Tables

**Table 1:** Hydrogen-bond geometry (Å, °)

*D*—H⋯*A*	*D*—H	H⋯*A*	*D*⋯*A*	*D*—H⋯*A*
C3—H3⋯N6^i^	0.93	2.59	3.261 (11)	129

Symmetry code: (i) 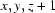.

**Fig. 1.**
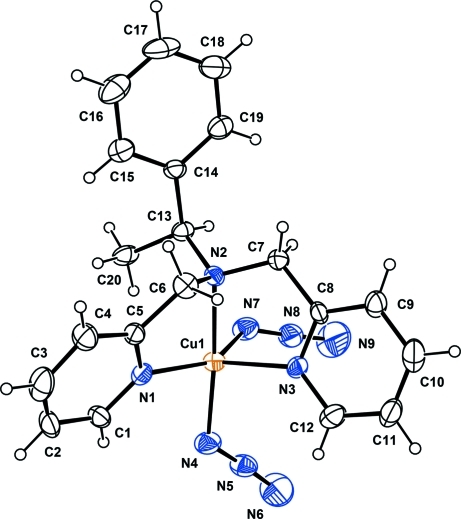
An ellipsoid plot (30% probability) of the title compound. Hydrogen atoms are drawn as small spheres of arbitrary radius.

**Fig. 2.**
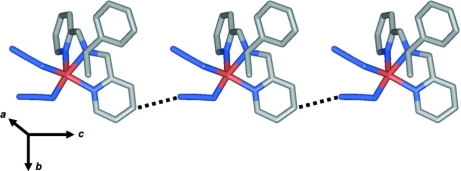
Perspective view of the title compound showing a one-dimensional chain formed by C—H···N hydrogen bonding interactions.

## supplementary crystallographic information

### Comment

Chiral complexes have attracted considerable interest because of their potential and practical applications, such as chiral recognition, chiral catalysis, and enantioselective sorption (Lehn, 1995; Seo *et al.*, 2000).  Very recently, a two-dimensional chiral open framework, [Ni(*L*^R,^*^R^*)]_3_[C_6_H_3_(COO)_3_]_2_.12H_2_O.CH_3_CN [*L^R^*^,^*^R^*=1,8-bis[(*R*)-*α*-methylbenzyl]-1,3,6,8,\ 10,13-\ hexaazacyclotetradecane] has been shown to have selective chiral recognition in *rac*-1,1'-bi-2-naphthol (Han *et al.*, 2008; Ryoo *et al.*, 2010).  Furthermore, a homochiral metal-organic framework composed of a cerium(III) ion and chiral organic building block has large chiral one-dimensional channels and exhibited excellent catalytic activity and high enantioselectivity for the asymmetric cyanosilylation of aromatic aldehydes (Dang *et al.*, 2010). Here, we report the synthesis and crystal structure of a five-coordinated Cu^II^ complex with (*S*)-1-phenyl-*N*,*N*-[bis(2-pyridyl)methyl]ethanamine (*S*-ppme), the title compound [Cu(*S*-ppme)(N_3_)_2_].

In the title compound (Fig. 1), the Cu^II^ ion is five-coordinated and shows a distorted square pyramidal geometry, the equatorial plane being defined by the three nitrogen atoms of the *S*-ppme ligand and one nitrogen atom of an azide ion. The coordination geometry is completed by the axial coordination of the nitrogen atom of the second azide anion. The Cu—*L*_eq_ bond lengths are in the range of 1.961 (6) and 2.178 (5) Å and the Cu—N_ax_ bond length is 1.978 (5) Å. Both azide ions are bonded in η^1^-fashion and fully delocalized. The bond angles around the copper atom range from 76.95 (12) to 165.48 (15)°. The packing structure involves a weak C—H···N hydrogen bonding interaction between the one pyridine group of the *S*-ppme ligand and an azide N atom of an adjacent complex unit (Table 1), giving a one-dimensional chain structure parallel to the *c* axis (Fig. 2).

### Experimental

(*S*)-1-phenyl-*N*,*N*-[bis(2-pyridyl)methyl]ethanamine (*S*-ppme) was prepared according to slightly modified literature procedure (Lucas *et al.*, 2009) except that (*S*)-(-)-*α*-methylbenzylamine instead of benzylamine (yield: 0.86 g, 60%). A mixture of MeCN and H_2_O (2:1, *v*/*v*, 3 ml) solution of CuCl_2_.2H_2_O (29 mg, 0.17 mmol) was added to an MeCN solution (3 ml) of *S*-ppme (51 mg, 0.17 mmol) and a MeOH solution (4 ml) of sodium azide (22 mg, 0.34 mmol). The resulting solution was stirred for 1 h at room temperature, resulting in a color change to blue-green. Diffusion of diethyl ether into the mixture gave green crystals of the title compound after a few days. These crystals were filtered and washed with diethyl ether and dried in air (yield: 42 mg, 56%).

### Refinement

All H atoms in the title compound were placed in geometrically idealized positions and constrained to ride on their parent atoms, with C—H distances of 0.93 (ring H atoms) and 0.96–0.98 Å (open chain H atoms), and with *U*_iso_(H) values of 1.2 or 1.5 times the equivalent anisotropic displacement parameters of the parent C atom.

### Crystal data

**Table:**

[Cu(N_3_)_2_(C_20_H_21_N_3_)]	*F*(000) = 466
*M**_r_* = 451.00	*D*_x_ = 1.451 Mg m^−^^3^
Monoclinic, *P*2_1_	Mo *K*α radiation, λ = 0.71073 Å
Hall symbol: P 2yb	Cell parameters from 2059 reflections
*a* = 6.9972 (12) Å	θ = 2.5–23.1°
*b* = 14.506 (3) Å	µ = 1.09 mm^−^^1^
*c* = 10.2828 (17) Å	*T* = 296 K
β = 98.413 (4)°	Plate, green
*V* = 1032.5 (3) Å^3^	0.23 × 0.19 × 0.04 mm
*Z* = 2	

### Data collection

**Table:**

Siemens SMART CCD diffractometer	4630 independent reflections
Radiation source: fine-focus sealed tube	2863 reflections with *I* > 2σ(*I*)
graphite	*R*_int_ = 0.049
φ and ω scans	θ_max_ = 28.3°, θ_min_ = 2.0°
Absorption correction: multi-scan (*SADABS*; Sheldrick, 1996)	*h* = −8→9
*T*_min_ = 0.749, *T*_max_ = 0.958	*k* = −19→16
7801 measured reflections	*l* = −13→8

### Refinement

**Table:**

Refinement on *F*^2^	Secondary atom site location: difference Fourier map
Least-squares matrix: full	Hydrogen site location: inferred from neighbouring sites
*R*[*F*^2^ > 2σ(*F*^2^)] = 0.058	H-atom parameters constrained
*wR*(*F*^2^) = 0.115	*w* = 1/[σ^2^(*F*_o_^2^) + 0.2609*P*] where *P* = (*F*_o_^2^ + 2*F*_c_^2^)/3
*S* = 1.09	(Δ/σ)_max_ < 0.001
4630 reflections	Δρ_max_ = 0.74 e Å^−^^3^
272 parameters	Δρ_min_ = −0.90 e Å^−^^3^
1 restraint	Absolute structure: Flack (1983), 1941 Friedel pairs
Primary atom site location: structure-invariant direct methods	Flack parameter: 0.02 (3)

### Special details

**Table:**

Geometry. All e.s.d.'s (except the e.s.d. in the dihedral angle between two l.s. planes) are estimated using the full covariance matrix. The cell e.s.d.'s are taken into account individually in the estimation of e.s.d.'s in distances, angles and torsion angles; correlations between e.s.d.'s in cell parameters are only used when they are defined by crystal symmetry. An approximate (isotropic) treatment of cell e.s.d.'s is used for estimating e.s.d.'s involving l.s. planes.
Refinement. Refinement of *F*^2^ against ALL reflections. The weighted *R*-factor *wR* and goodness of fit *S* are based on *F*^2^, conventional *R*-factors *R* are based on *F*, with *F* set to zero for negative *F*^2^. The threshold expression of *F*^2^ > σ(*F*^2^) is used only for calculating *R*-factors(gt) *etc*. and is not relevant to the choice of reflections for refinement. *R*-factors based on *F*^2^ are statistically about twice as large as those based on *F*, and *R*- factors based on ALL data will be even larger.

### Fractional atomic coordinates and isotropic or equivalent isotropic displacement parameters (Å^2^)

**Table:**

	*x*	*y*	*z*	*U*_iso_*/*U*_eq_	
Cu1	0.97299 (8)	0.03627 (6)	0.09599 (6)	0.03987 (19)	
N1	0.9182 (6)	0.1005 (4)	0.2601 (5)	0.0397 (13)	
N2	1.1219 (6)	−0.0557 (3)	0.2383 (5)	0.0352 (12)	
N3	0.7954 (6)	−0.0873 (4)	0.0636 (5)	0.0391 (13)	
N4	0.8063 (8)	0.1251 (5)	−0.0106 (6)	0.0594 (18)	
N5	0.7529 (8)	0.1156 (4)	−0.1229 (7)	0.0571 (16)	
N6	0.6948 (12)	0.1098 (6)	−0.2324 (7)	0.113 (3)	
N7	1.1556 (7)	0.0125 (5)	−0.0299 (6)	0.056 (2)	
N8	1.1260 (9)	−0.0280 (5)	−0.1291 (7)	0.0662 (19)	
N9	1.1054 (13)	−0.0689 (8)	−0.2243 (9)	0.150 (5)	
C1	0.8573 (8)	0.1875 (5)	0.2688 (7)	0.0476 (17)	
H1	0.8397	0.2237	0.1934	0.057*	
C2	0.8202 (9)	0.2249 (5)	0.3835 (8)	0.061 (2)	
H2	0.7774	0.2854	0.3864	0.073*	
C3	0.8470 (10)	0.1716 (7)	0.4955 (8)	0.071 (2)	
H3	0.8182	0.1951	0.5745	0.085*	
C4	0.9162 (9)	0.0839 (5)	0.4894 (7)	0.055 (2)	
H4	0.9390	0.0474	0.5645	0.066*	
C5	0.9513 (7)	0.0509 (5)	0.3707 (5)	0.0375 (17)	
C6	1.0194 (8)	−0.0470 (5)	0.3535 (6)	0.0433 (16)	
H6A	1.1050	−0.0656	0.4319	0.052*	
H6B	0.9088	−0.0881	0.3428	0.052*	
C7	1.0910 (8)	−0.1481 (4)	0.1814 (6)	0.0412 (15)	
H7A	1.1169	−0.1934	0.2512	0.049*	
H7B	1.1829	−0.1583	0.1207	0.049*	
C8	0.8881 (8)	−0.1635 (4)	0.1091 (6)	0.0376 (14)	
C9	0.8111 (8)	−0.2496 (5)	0.0895 (6)	0.0518 (18)	
H9	0.8782	−0.3010	0.1257	0.062*	
C10	0.6315 (10)	−0.2587 (6)	0.0148 (7)	0.066 (2)	
H10	0.5770	−0.3167	−0.0025	0.079*	
C11	0.5346 (10)	−0.1809 (6)	−0.0334 (6)	0.059 (2)	
H11	0.4125	−0.1853	−0.0828	0.071*	
C12	0.6200 (8)	−0.0968 (5)	−0.0079 (6)	0.0535 (19)	
H12	0.5541	−0.0442	−0.0413	0.064*	
C13	1.3354 (8)	−0.0333 (5)	0.2654 (6)	0.0376 (17)	
H13	1.3882	−0.0501	0.1855	0.045*	
C14	1.4474 (7)	−0.0887 (5)	0.3751 (6)	0.0397 (15)	
C15	1.4879 (9)	−0.0563 (5)	0.5031 (6)	0.0551 (18)	
H15	1.4398	0.0005	0.5249	0.066*	
C16	1.6004 (11)	−0.1086 (6)	0.5992 (7)	0.072 (2)	
H16	1.6268	−0.0864	0.6848	0.087*	
C17	1.6718 (10)	−0.1919 (7)	0.5691 (9)	0.077 (3)	
H17	1.7478	−0.2257	0.6341	0.092*	
C18	1.6329 (10)	−0.2265 (6)	0.4442 (8)	0.068 (2)	
H18	1.6803	−0.2838	0.4238	0.082*	
C19	1.5211 (8)	−0.1742 (5)	0.3486 (6)	0.0508 (18)	
H19	1.4948	−0.1975	0.2635	0.061*	
C20	1.3706 (8)	0.0698 (5)	0.2839 (8)	0.050 (2)	
H20A	1.3169	0.0905	0.3595	0.075*	
H20B	1.3102	0.1021	0.2074	0.075*	
H20C	1.5070	0.0817	0.2966	0.075*	

### Atomic displacement parameters (Å^2^)

**Table:**

	*U*^11^	*U*^22^	*U*^33^	*U*^12^	*U*^13^	*U*^23^
Cu1	0.0360 (3)	0.0420 (4)	0.0411 (4)	0.0039 (5)	0.0038 (2)	0.0023 (5)
N1	0.034 (3)	0.048 (4)	0.036 (3)	−0.002 (2)	0.001 (2)	−0.005 (3)
N2	0.033 (3)	0.029 (3)	0.044 (3)	0.000 (2)	0.005 (2)	−0.001 (2)
N3	0.029 (2)	0.042 (4)	0.046 (3)	−0.004 (2)	0.005 (2)	−0.005 (3)
N4	0.063 (4)	0.070 (5)	0.042 (4)	0.017 (3)	−0.002 (3)	0.001 (3)
N5	0.062 (4)	0.058 (5)	0.051 (4)	0.014 (3)	0.006 (3)	0.012 (3)
N6	0.169 (8)	0.112 (7)	0.050 (5)	0.037 (6)	−0.012 (5)	0.006 (4)
N7	0.044 (3)	0.074 (6)	0.052 (3)	0.008 (3)	0.009 (2)	−0.003 (3)
N8	0.064 (4)	0.075 (5)	0.057 (4)	0.022 (3)	0.002 (3)	−0.017 (4)
N9	0.134 (8)	0.207 (12)	0.103 (7)	0.055 (7)	−0.002 (6)	−0.088 (8)
C1	0.039 (3)	0.039 (5)	0.065 (5)	0.006 (3)	0.008 (3)	−0.009 (3)
C2	0.052 (4)	0.052 (6)	0.079 (6)	0.004 (4)	0.013 (4)	−0.032 (5)
C3	0.059 (5)	0.099 (8)	0.058 (5)	−0.008 (5)	0.021 (4)	−0.034 (5)
C4	0.054 (4)	0.062 (6)	0.052 (4)	0.005 (3)	0.015 (3)	−0.010 (3)
C5	0.031 (2)	0.043 (5)	0.039 (3)	0.002 (3)	0.007 (2)	−0.008 (4)
C6	0.043 (4)	0.042 (5)	0.048 (4)	−0.009 (3)	0.017 (3)	0.007 (3)
C7	0.034 (3)	0.035 (4)	0.053 (4)	0.001 (3)	0.002 (3)	−0.004 (3)
C8	0.035 (3)	0.027 (4)	0.051 (4)	−0.001 (3)	0.008 (3)	−0.012 (3)
C9	0.037 (3)	0.052 (5)	0.067 (5)	−0.005 (3)	0.011 (3)	−0.021 (4)
C10	0.055 (5)	0.065 (6)	0.081 (6)	−0.016 (4)	0.023 (4)	−0.032 (5)
C11	0.048 (4)	0.068 (7)	0.058 (5)	−0.012 (4)	−0.002 (3)	−0.019 (4)
C12	0.038 (3)	0.067 (6)	0.054 (4)	−0.002 (4)	0.002 (3)	−0.003 (4)
C13	0.032 (3)	0.038 (5)	0.042 (4)	−0.003 (3)	0.002 (3)	0.006 (3)
C14	0.029 (3)	0.039 (4)	0.049 (4)	0.002 (3)	−0.001 (3)	0.003 (3)
C15	0.053 (4)	0.058 (5)	0.052 (4)	0.003 (4)	−0.001 (3)	0.003 (4)
C16	0.076 (5)	0.085 (8)	0.051 (5)	−0.017 (5)	−0.007 (4)	0.020 (5)
C17	0.055 (5)	0.078 (8)	0.089 (7)	−0.003 (4)	−0.017 (4)	0.048 (5)
C18	0.050 (4)	0.059 (6)	0.091 (6)	0.003 (4)	−0.002 (4)	0.020 (5)
C19	0.041 (3)	0.057 (5)	0.053 (4)	−0.004 (3)	0.002 (3)	0.003 (3)
C20	0.027 (3)	0.054 (6)	0.067 (5)	−0.005 (3)	−0.003 (3)	0.010 (3)

### Geometric parameters (Å, °)

**Table:**

Cu1—N4	1.961 (6)	C7—H7A	0.9700
Cu1—N7	1.978 (5)	C7—H7B	0.9700
Cu1—N1	2.013 (5)	C8—C9	1.363 (8)
Cu1—N2	2.135 (5)	C9—C10	1.380 (8)
Cu1—N3	2.178 (5)	C9—H9	0.9300
N1—C5	1.337 (7)	C10—C11	1.371 (10)
N1—C1	1.339 (8)	C10—H10	0.9300
N2—C7	1.466 (7)	C11—C12	1.367 (9)
N2—C6	1.477 (6)	C11—H11	0.9300
N2—C13	1.514 (7)	C12—H12	0.9300
N3—C8	1.332 (7)	C13—C14	1.508 (9)
N3—C12	1.342 (7)	C13—C20	1.522 (8)
N4—N5	1.169 (8)	C13—H13	0.9800
N5—N6	1.144 (7)	C14—C19	1.386 (8)
N7—N8	1.168 (7)	C14—C15	1.388 (8)
N8—N9	1.137 (8)	C15—C16	1.395 (9)
C1—C2	1.357 (9)	C15—H15	0.9300
C1—H1	0.9300	C16—C17	1.360 (11)
C2—C3	1.377 (10)	C16—H16	0.9300
C2—H2	0.9300	C17—C18	1.369 (10)
C3—C4	1.366 (10)	C17—H17	0.9300
C3—H3	0.9300	C18—C19	1.389 (9)
C4—C5	1.366 (8)	C18—H18	0.9300
C4—H4	0.9300	C19—H19	0.9300
C5—C6	1.517 (10)	C20—H20A	0.9600
C6—H6A	0.9700	C20—H20B	0.9600
C6—H6B	0.9700	C20—H20C	0.9600
C7—C8	1.520 (7)		
			
N4—Cu1—N7	97.9 (2)	C8—C7—H7A	108.8
N4—Cu1—N1	89.6 (2)	N2—C7—H7B	108.8
N7—Cu1—N1	148.2 (2)	C8—C7—H7B	108.8
N4—Cu1—N2	169.7 (2)	H7A—C7—H7B	107.7
N7—Cu1—N2	92.4 (2)	N3—C8—C9	123.2 (5)
N1—Cu1—N2	81.3 (2)	N3—C8—C7	115.0 (5)
N4—Cu1—N3	100.1 (2)	C9—C8—C7	121.8 (6)
N7—Cu1—N3	99.5 (2)	C8—C9—C10	118.6 (7)
N1—Cu1—N3	109.55 (19)	C8—C9—H9	120.7
N2—Cu1—N3	78.53 (19)	C10—C9—H9	120.7
C5—N1—C1	117.9 (6)	C11—C10—C9	118.9 (7)
C5—N1—Cu1	115.7 (5)	C11—C10—H10	120.5
C1—N1—Cu1	126.4 (4)	C9—C10—H10	120.5
C7—N2—C6	109.7 (5)	C12—C11—C10	119.1 (7)
C7—N2—C13	110.7 (4)	C12—C11—H11	120.5
C6—N2—C13	114.5 (5)	C10—C11—H11	120.5
C7—N2—Cu1	105.6 (3)	N3—C12—C11	122.4 (7)
C6—N2—Cu1	104.5 (3)	N3—C12—H12	118.8
C13—N2—Cu1	111.2 (4)	C11—C12—H12	118.8
C8—N3—C12	117.8 (6)	C14—C13—N2	114.5 (5)
C8—N3—Cu1	113.0 (4)	C14—C13—C20	111.9 (6)
C12—N3—Cu1	128.6 (5)	N2—C13—C20	111.8 (6)
N5—N4—Cu1	123.6 (5)	C14—C13—H13	106.0
N6—N5—N4	176.8 (8)	N2—C13—H13	106.0
N8—N7—Cu1	127.6 (5)	C20—C13—H13	106.0
N9—N8—N7	176.9 (8)	C19—C14—C15	117.3 (6)
N1—C1—C2	122.5 (7)	C19—C14—C13	119.9 (6)
N1—C1—H1	118.7	C15—C14—C13	122.7 (6)
C2—C1—H1	118.7	C14—C15—C16	120.3 (7)
C1—C2—C3	118.8 (8)	C14—C15—H15	119.9
C1—C2—H2	120.6	C16—C15—H15	119.9
C3—C2—H2	120.6	C17—C16—C15	120.7 (8)
C4—C3—C2	119.4 (7)	C17—C16—H16	119.7
C4—C3—H3	120.3	C15—C16—H16	119.7
C2—C3—H3	120.3	C16—C17—C18	120.7 (7)
C5—C4—C3	118.5 (7)	C16—C17—H17	119.7
C5—C4—H4	120.7	C18—C17—H17	119.7
C3—C4—H4	120.7	C17—C18—C19	118.6 (8)
N1—C5—C4	122.7 (7)	C17—C18—H18	120.7
N1—C5—C6	115.0 (5)	C19—C18—H18	120.7
C4—C5—C6	122.2 (6)	C14—C19—C18	122.4 (7)
N2—C6—C5	111.8 (5)	C14—C19—H19	118.8
N2—C6—H6A	109.3	C18—C19—H19	118.8
C5—C6—H6A	109.3	C13—C20—H20A	109.5
N2—C6—H6B	109.3	C13—C20—H20B	109.5
C5—C6—H6B	109.3	H20A—C20—H20B	109.5
H6A—C6—H6B	107.9	C13—C20—H20C	109.5
N2—C7—C8	113.7 (5)	H20A—C20—H20C	109.5
N2—C7—H7A	108.8	H20B—C20—H20C	109.5
			
N4—Cu1—N1—C5	159.9 (4)	Cu1—N1—C5—C4	−178.2 (4)
N7—Cu1—N1—C5	−95.6 (6)	C1—N1—C5—C6	179.8 (5)
N2—Cu1—N1—C5	−15.2 (4)	Cu1—N1—C5—C6	−1.7 (6)
N3—Cu1—N1—C5	59.3 (4)	C3—C4—C5—N1	−0.9 (9)
N4—Cu1—N1—C1	−21.8 (5)	C3—C4—C5—C6	−177.1 (6)
N7—Cu1—N1—C1	82.7 (6)	C7—N2—C6—C5	−148.8 (5)
N2—Cu1—N1—C1	163.1 (5)	C13—N2—C6—C5	86.0 (6)
N3—Cu1—N1—C1	−122.4 (5)	Cu1—N2—C6—C5	−35.9 (5)
N4—Cu1—N2—C7	114.6 (13)	N1—C5—C6—N2	27.1 (7)
N7—Cu1—N2—C7	−68.0 (4)	C4—C5—C6—N2	−156.5 (5)
N1—Cu1—N2—C7	143.3 (4)	C6—N2—C7—C8	72.5 (6)
N3—Cu1—N2—C7	31.2 (3)	C13—N2—C7—C8	−160.1 (5)
N4—Cu1—N2—C6	−1.1 (15)	Cu1—N2—C7—C8	−39.6 (5)
N7—Cu1—N2—C6	176.3 (4)	C12—N3—C8—C9	−1.9 (9)
N1—Cu1—N2—C6	27.6 (3)	Cu1—N3—C8—C9	−174.1 (5)
N3—Cu1—N2—C6	−84.5 (4)	C12—N3—C8—C7	176.1 (5)
N4—Cu1—N2—C13	−125.2 (13)	Cu1—N3—C8—C7	3.9 (6)
N7—Cu1—N2—C13	52.2 (4)	N2—C7—C8—N3	24.9 (7)
N1—Cu1—N2—C13	−96.5 (4)	N2—C7—C8—C9	−157.1 (5)
N3—Cu1—N2—C13	151.4 (4)	N3—C8—C9—C10	2.5 (9)
N4—Cu1—N3—C8	170.0 (4)	C7—C8—C9—C10	−175.4 (6)
N7—Cu1—N3—C8	70.2 (4)	C8—C9—C10—C11	−2.0 (10)
N1—Cu1—N3—C8	−96.7 (4)	C9—C10—C11—C12	1.0 (10)
N2—Cu1—N3—C8	−20.4 (4)	C8—N3—C12—C11	0.8 (9)
N4—Cu1—N3—C12	−1.1 (6)	Cu1—N3—C12—C11	171.6 (5)
N7—Cu1—N3—C12	−101.0 (5)	C10—C11—C12—N3	−0.4 (10)
N1—Cu1—N3—C12	92.1 (5)	C7—N2—C13—C14	−68.9 (7)
N2—Cu1—N3—C12	168.5 (5)	C6—N2—C13—C14	55.8 (7)
N7—Cu1—N4—N5	43.4 (7)	Cu1—N2—C13—C14	174.0 (4)
N1—Cu1—N4—N5	−167.6 (6)	C7—N2—C13—C20	162.5 (6)
N2—Cu1—N4—N5	−139.2 (11)	C6—N2—C13—C20	−72.8 (7)
N3—Cu1—N4—N5	−57.8 (6)	Cu1—N2—C13—C20	45.4 (7)
N4—Cu1—N7—N8	−71.0 (7)	N2—C13—C14—C19	86.4 (7)
N1—Cu1—N7—N8	−173.1 (6)	C20—C13—C14—C19	−145.1 (6)
N2—Cu1—N7—N8	109.5 (7)	N2—C13—C14—C15	−96.2 (7)
N3—Cu1—N7—N8	30.7 (7)	C20—C13—C14—C15	32.3 (9)
C5—N1—C1—C2	−3.1 (9)	C13—C14—C15—C16	−176.8 (6)
Cu1—N1—C1—C2	178.7 (5)	C15—C16—C17—C18	−0.7 (12)
N1—C1—C2—C3	0.3 (10)	C16—C17—C18—C19	0.8 (11)
C1—C2—C3—C4	2.2 (10)	C15—C14—C19—C18	−0.6 (9)
C2—C3—C4—C5	−1.9 (10)	C13—C14—C19—C18	177.0 (6)
C1—N1—C5—C4	3.4 (8)		

### Hydrogen-bond geometry (Å, °)

**Table:**

*D*—H···*A*	*D*—H	H···*A*	*D*···*A*	*D*—H···*A*
C3—H3···N6^i^	0.93	2.59	3.261 (11)	129

Symmetry codes: (i) *x*, *y*, *z*+1.
